# Spiritual places: Spatial recognition of Tibetan Buddhist spiritual perception

**DOI:** 10.1371/journal.pone.0301087

**Published:** 2024-05-23

**Authors:** Dongzhu Gadan, Zaisheng Zhang

**Affiliations:** College of Management and Economics, Tianjin University, Tianjin, China; Universidad de Sevilla Escuela Técnica Superior de Arquitectura: Universidad de Sevilla Escuela Tecnica Superior de Arquitectura, SPAIN

## Abstract

Tibetan Buddhism, as an indigenous religion, has a significant and far-reaching influence in the Tibetan areas of China. This study, focusing on Lhasa, explores the integration of Tibetan Buddhist spiritual perceptions within urban spaces. Employing a novel approach that combines street view data and deep learning technology, the research aims to identify and map the spatial distribution of Tibetan Buddhist spiritual sites against the backdrop of the urban landscape. Our analysis reveals a notable concentration of these spiritual places near urban architectural and cultural heritage areas, highlighting the profound connection between residents’ cultural life and spiritual practices. Despite challenges posed by modern urbanisation, these spiritual sites demonstrate resilience and adaptability, continuing to serve as cultural and spiritual pillars of the Tibetan Buddhist community. This study contributes to the fields of urban planning, religious studies, and digital humanities by demonstrating the potential of technology in examining the impact of urban development on cultural and religious landscapes. The research underscores the importance of protecting and integrating spaces of spiritual perception in urban development planning. It shows that safeguarding these spaces is crucial not only for cultural heritage preservation but also for achieving sustainable urban development and social harmony. This study opens new avenues for interdisciplinary research, advocating for a deeper understanding of the dynamic relationship between urban development and spiritual spaces from psychological, sociological, and environmental science perspectives. As urban landscapes evolve, the study emphasises the need to maintain a balance between material sustainability and cultural and spiritual richness in urban planning.

## Introduction

Tibetan Buddhism is not merely a religious faith or philosophical system, but a multidimensional cultural and spiritual phenomenon. Its influence is extensive and profound, spanning multiple domains such as society, art, psychology, and geographical space [1]. Compared to the superficial understanding common in the Western academic community and the general populace—such as monks, temples, and rituals—in the East, particularly in Tibetan-influenced regions and their surrounding cultural spheres, the influence of Tibetan Buddhism extends far beyond these visible symbols [2]. It constructs an ineffable "spiritual place" in people’s minds, forming not only the core of individual and collective spiritual pursuits [3] but also a symbol of cultural identity and social cohesion. The concept of this "spiritual place" provides a framework for understanding (Walter, 2009), aiding in the exploration of how Tibetan Buddhism influences and shapes people’s inner spaces at a deeper level, thereby further affecting their cultural and social concepts [4].

In the context of globalization and rapid urbanization, the transformation and shaping of physical spaces often garner the most attention. The emergence of new buildings and the reconstruction or demolition of old communities are direct manifestations of urban development [5, 6]. However, parallel to this change in physical space is the evolution of mental spaces or "spiritual places" [7]. Especially under the profound cultural backdrop of Tibetan Buddhism, spiritual places do not always correspond directly with physical spaces. An undeveloped plot of land, a dilapidated temple, or even an ordinary road can become a "place" of significant spiritual meaning [8]. In the process of urban development, these spiritual places face the risk of being marginalized or disappearing [9, 10]. On one hand, the push towards modernization and commercialization may lead to the alteration or destruction of the physical structures of traditional spiritual places [11]. On the other hand, the social and cultural value of spiritual places may be overlooked or diminished due to a lack of understanding and respect for their deeper meanings [12, 13]. This phenomenon is particularly evident in Tibetan-influenced areas and is also reflected in various cities and cultures around the globe [14–16].

However, in this seemingly irreversible process, the spiritual places of Tibetan Buddhism exhibit remarkable resilience and adaptability [17, 18]. Even in situations where physical spaces are significantly disrupted, spiritual places can still be preserved or transformed in some manner [19, 20]. For instance, in some rapidly developing cities, traditional temples may be replaced by skyscrapers, but certain elements or symbolic meanings of the temples may reappear in new forms or through new mediums [21, 22]. The way this interactive relationship shapes or redefines our perception and experience of “place” warrants in-depth exploration [23, 24].

The study and understanding of "space" have historically focused primarily on physical, geographical, or socio-economic dimensions [25–27]. While these studies have provided us with a wealth of perspectives and theoretical foundations [28, 29], they often overlook or undervalue the importance of "mental space" or "spiritual place". Even in research that involves cultural or psychological factors, mental space is usually seen as a secondary or subsidiary concept, with few dedicated studies exploring it comprehensively and deeply [30, 31]. This limitation is evident in both Western and Eastern academic circles, but more so in the latter. In most Eastern cultures, especially within Tibetan Buddhist culture, mental space is not only as important as physical space, but in some cases, even more crucial. This type of space influences not only the psychological state of individuals and communities but also their relationships with the environment, others, and even higher-dimensional existences. Past research has tended to understand mental space from the perspective of a single cultural or religious background, overlooking the diversity and interactivity of mental spaces across different cultures and belief systems [32, 33]. In today’s world of globalization and multicultural integration, this singular perspective is inadequate for understanding and explaining complex realities. For instance, in projects aimed at preserving cultural heritage and promoting sustainable development, the neglect of local mental spaces often leads to project failures or adverse outcomes. The importance of mental space is underestimated in both natural and built environments in most cases, leading to an incomplete understanding of local culture and social structures [34, 35].

With the rapid evolution of digitalization and artificial intelligence technologies [36, 37], more precise and flexible tools are now available for decoding the complex interactions between physical and mental spaces [38, 39]. Particularly in the field of urban management and planning, optimizing and protecting spiritual places has gradually emerged as a crucial issue. This study employs street view image recognition technology and integrates it with methodologies from human geography and cultural psychology, to systematically explore the tangible manifestations of Tibetan Buddhism’s spiritual perceptual space in actual urban layouts. This interdisciplinary approach not only aids in comprehensively identifying these often marginalized but significant spiritual places at both macro and micro levels [40] but also generates more actionable information and data, thereby facilitating ongoing optimization in urban management and planning. Specifically, street view image recognition can accurately capture the physical location and spatial distribution of Tibetan Buddhist elements and symbols—such as prayer flags, mani stone piles, and temples—in the urban environment. This allows for an in-depth analysis of the interactions and impacts between these spiritual places and their surrounding environments—including commercial, residential, and natural spaces. Such analysis not only provides powerful decision-making support for urban managers and policymakers [41] but also effectively protects and maintains places rich in cultural and spiritual value. For example, the application of street view image recognition might reveal a Tibetan Buddhist spiritual place within a commercial district that has been overlooked. Once identified, planners can preserve or emphasize its cultural and spiritual significance in future development plans, or even consider promoting its cultural and spiritual revival through the addition of corresponding cultural activities or educational programs.

Overall, this study, by organically integrating street view image recognition technology with the humanities and social sciences, aims not only to deepen our understanding of Tibetan Buddhist spiritual places but also strives to promote their protection and optimization in modern urban environments. This is done with the goal of fostering a symbiosis between cultural heritage and contemporary development.

## Data

**Fig 1:**
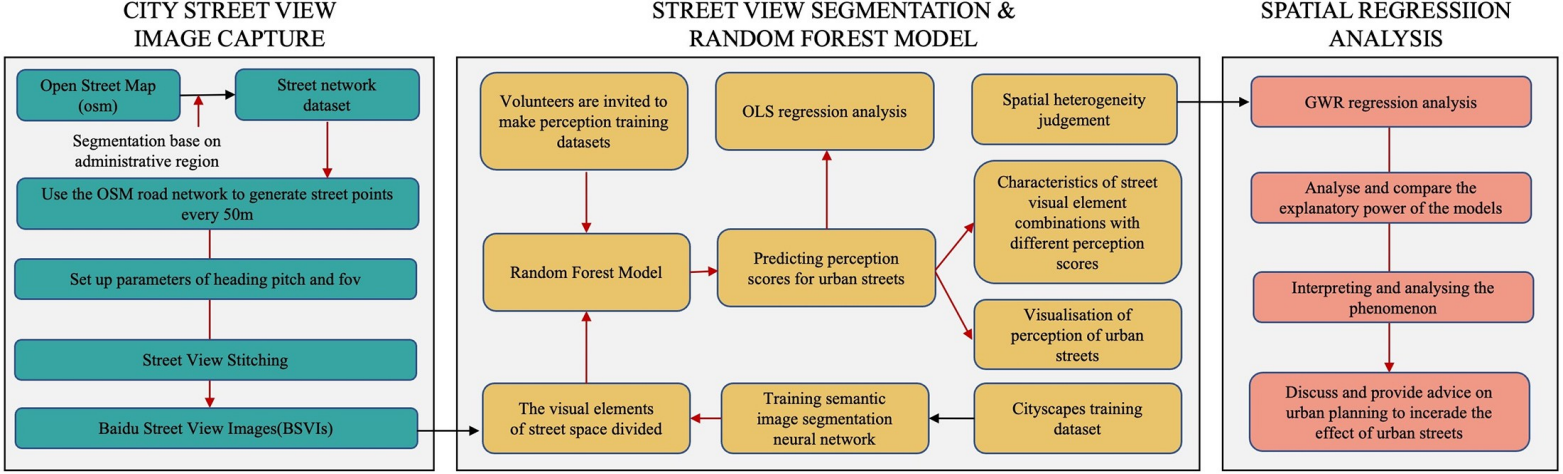
Overview of our workflow.

Fig 1 presents a conceptual framework for the exploration of restorative perceptions in urban streets. The process begins by downloading the road network of the study area based on administrative boundaries from the OSM website. Street view points are then selected every 50 meters along the road network, simulating a pedestrian’s perspective to adjust the parameters for street view image collection. The Baidu Maps API is employed to gather large-scale urban street view data. Four street view images from the same point are stitched together to obtain a comprehensive view of each location in the study area. The next step involves training an image semantic segmentation model to obtain visual element data from urban street views. Concurrently, volunteers are invited to rate some of these street views based on a Tibetan Buddhist perception scale. The ratings are then combined with the street view visual elements to create a dataset for machine learning. This dataset is imported into a random forest model to predict Tibetan Buddhist perceptions across all street view images in the study area. The perceived scores are then correlated with urban Points of Interest (POI) features for analysis. The results of this analysis are used to make assessments about the influencing factors. Finally, the study discusses and provides recommendations for urban planners on enhancing the perception of Tibetan Buddhism in urban settings.

### Research area

**Fig 2:**
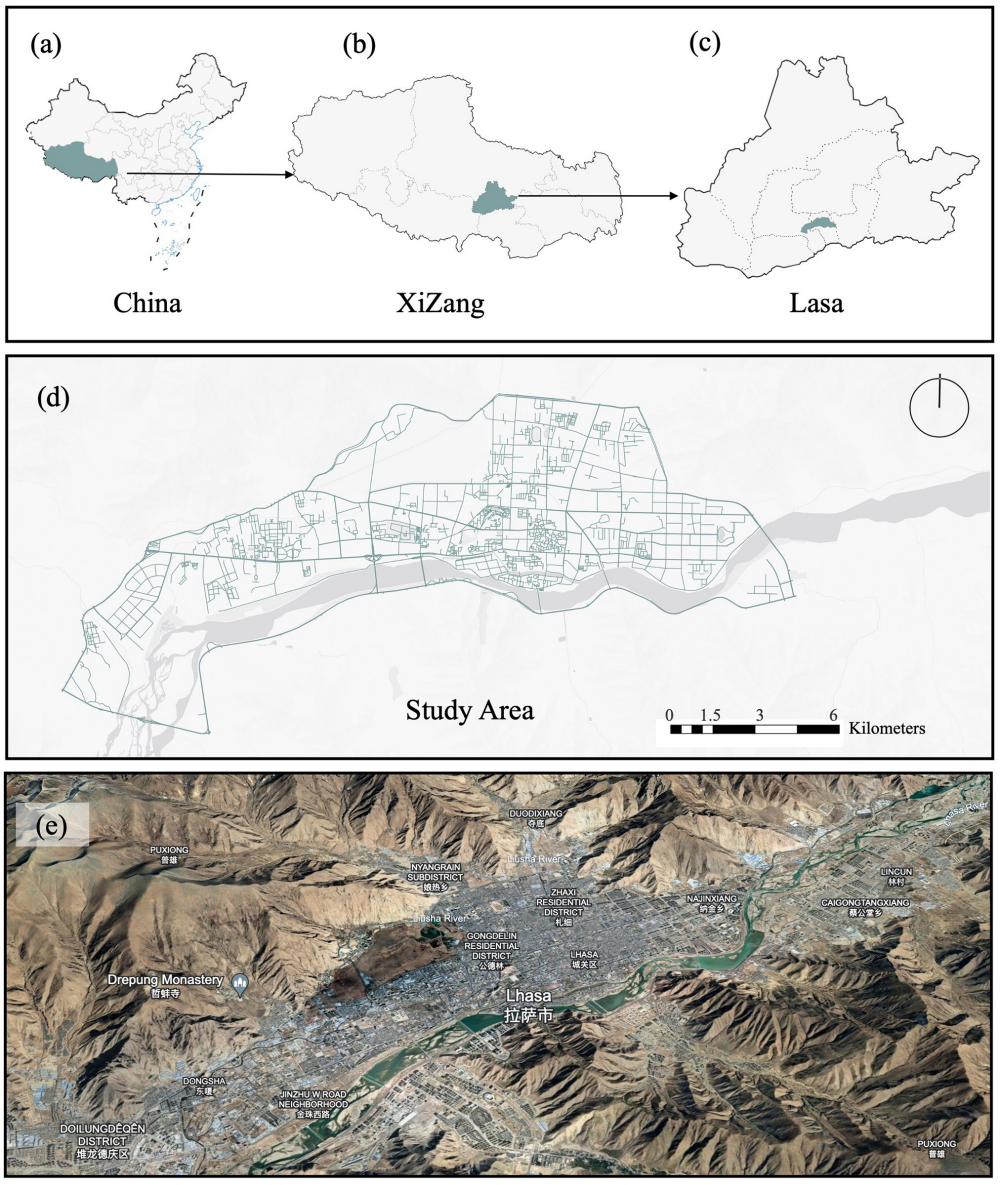
Study area. (a) China; (b) Xizang; (c) Lasa; (d) Study Area; (e) Satellite Map.

**Fig 3:**
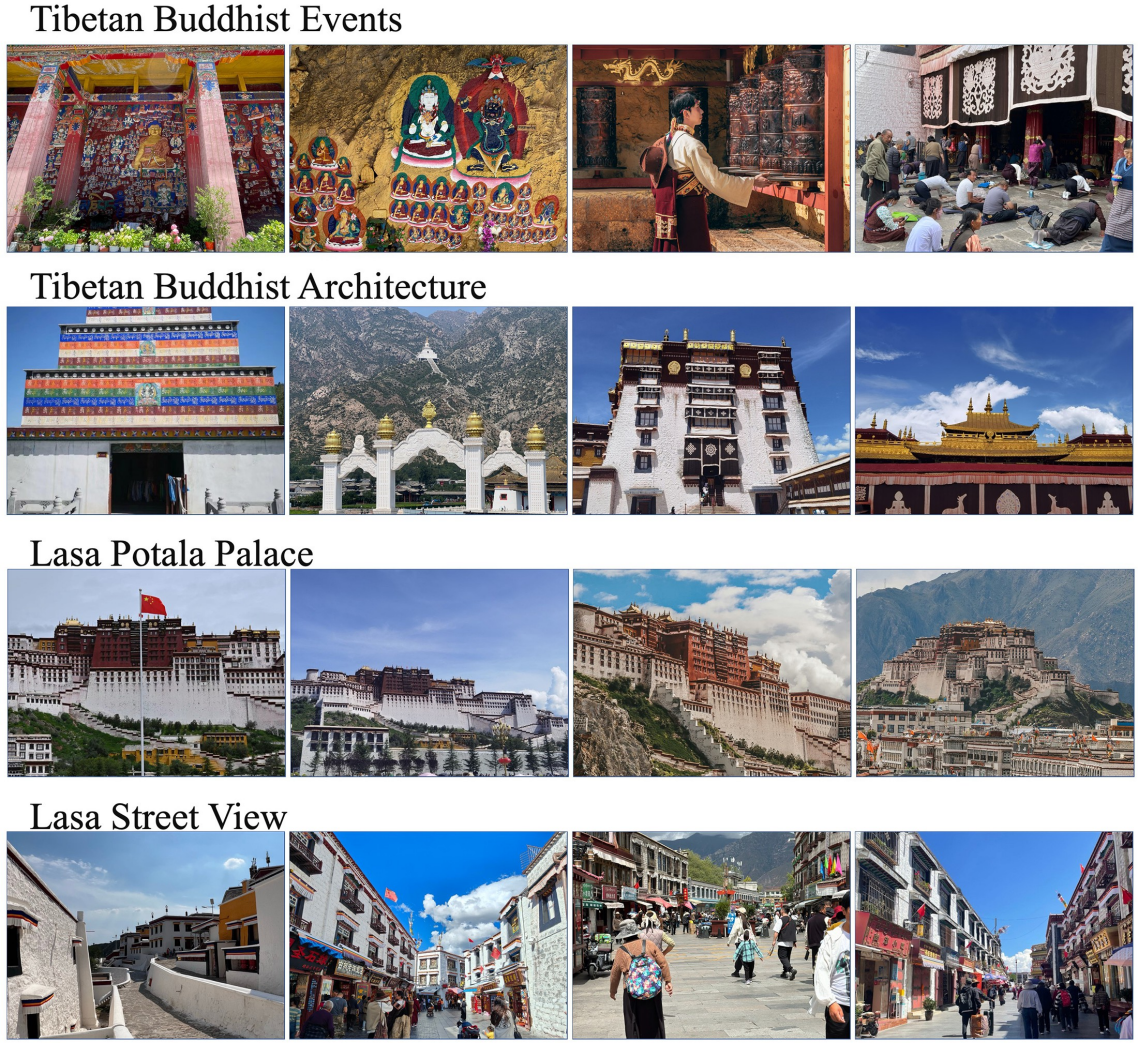
Study area Tibetan Buddhist elements of the real scene map.

The focus of this study is centered on Lhasa, the capital of the Tibet Autonomous Region in China, as detailed in Fig 2. Lhasa has a population of approximately 330,000, accounting for about 20% of the total population of the Tibet Autonomous Region. Situated at an altitude of 3,650 meters, Lhasa is surrounded by several mountain ranges, including the famous Himalayas. The Lhasa River runs through the city, creating a geographical diversity that offers a rich backdrop for studying the heterogeneity of perceptual spaces. Lhasa is not only the political and economic center of the Tibet Autonomous Region but also the spiritual heartland of Tibetan Buddhism. It houses several important Buddhist holy sites, including the Potala Palace, Jokhang Temple, and Sera Monastery. These temples and cultural sites have a significant impact on the perception of Tibetan Buddhism, as they are symbols of faith and spaces for physical and mental restoration. In Fig 3, we present elements of Tibetan Buddhism, Tibetan Buddhist architecture, the Potala Palace, and urban streetscapes. These photographs taken on-site provided by us greatly assist readers in understanding the research area.

Due to Lhasa’s profound religious culture and complex geographical environment, empirical research on the perception of Tibetan Buddhism here holds particular significance. This not only provides references for Lhasa itself in terms of urban planning and cultural preservation but also offers unique insights for other regions with rich religious and cultural backgrounds. Therefore, this study selects Lhasa as its base, focusing on exploring the manifestation and impact of Tibetan Buddhist perception in the city’s streets and temple spaces.

### Collecting Baidu street view image data

In the study area of Lhasa city, street view images were collected along roads to mimic the human perspective, with the aim of assessing Tibetan Buddhist perception in the region. The use of large-scale street view image data for analyzing urban street quality and the urban environment is becoming increasingly common [42]. Street view data platforms offer users the ability to perceive and observe the urban environment from a human-centric viewpoint [43]. These platforms not only provide street view browsing services to online users but also publish Application Programming Interfaces (APIs) that allow for quantitative, large-scale downloading of street view data. Thus, street view images represent the architectural environment of urban streets and serve as the basis for measuring perception.

To collect street view images, the road network provided by OpenStreetMap was used, and geographic coordinates for street view collection were generated at 50-meter intervals. The Baidu Maps API interface was then employed to download the latest street view image data at each point, from angles of 0°, 90°, 180°, and 270°. Specifically, 360° images of street views were collected at each coordinate point, with each point consisting of 4 images (0–90°, 90–180°, 180–270°, 270–360°). The following URL was used to obtain image IDs for specific locations: http://api.map.baidu.com/panorama/v2?ak=API_Key&width=600&height=400&location=LAT,LON&pitch=20&fov=90&heading=0/90/180/270.

In this URL, LAT and LON represent latitude and longitude, respectively; FOV determines the horizontal field of view of the image; HEADING indicates the compass heading of the camera; PITCH represents the upward or downward angle of the camera relative to the street view vehicle; and the API key is the credential required to validate requests. Street view image data were downloaded from four angles at each coordinate point, resulting in a total of 11,393 street view images for Lhasa city. The four street view images from each coordinate point were stitched into one panoramic image, resulting in 45,572 processed street view images.

### Tibetan Buddhist perception label data

In the process of creating Tibetan Buddhist perception label data, a street view image rating program was deployed on a Tencent Cloud server, facilitating researchers to access the server and conduct perception rating experiments from any computer device. This study adopts a human-centric perspective for evaluating perceptions. To measure the perception of Lhasa city’s urban streets, we recruited 30 volunteers comprising university students and working personnel, with a gender ratio of approximately 1:1 and ages ranging from 18 to 57 years. Volunteers could rate the urban Tibetan Buddhist perception of Lhasa’s street views through the Tencent Cloud server. All the volunteers understood what the experiment was about and agreed to collect the data for the study. The volunteer experiment was conducted between October and December 2022.

Referring to the perception factor scale (RCS) developed by Laumann et al. [44], a Tibetan Buddhist perception scale was created to guide volunteers in contemplating their perceptions. The perception questionnaire in this study, based on the perception factor scale, was designed to survey people’s Tibetan Buddhist perceptual experiences when viewing street images. The final rating represents the volunteers’ genuine subjective feelings of restorativeness for each street view. The questionnaire used in this study includes 50 randomly selected street view images and 16 questions corresponding to each image (Tab.1), evaluating the restorative perception of the streets from four aspects: religion, sacred, respect, and commandment.

Considering the time standards for rating individual images in other studies, volunteers were informed to observe each image for no less than 10 seconds [45, 46]. During this time, volunteers would perceive the street view image and score their answers using a 7-point Likert scale, where 0–6 points indicate the degree of agreement and approval of the content of the question. Specifically, a score of 0 was given if the description of the question was very inconsistent with the feelings when viewing the street scene, and a score of 6 if it was very consistent. The final score for each street view image was the average of the scores for the 16 questions (Table 1).

**Table 1:** Tibetan Buddhism perception scale.

ART Divisor	RCS questions
Religion	Here, I am able to find a connection with a higher power, such as a deity or the universe
	I feel a presence here that transcends everyday life
	In this environment, I feel spiritually nourished
	I believe this place can help me gain a deeper understanding of life
Sacred	I believe this environment is sacred and inviolable
	There is a kind of awe-inspiring beauty or power here
	In this place, I can feel a divine tranquility
	I think everything here is related to God or a higher power
Respect	I feel a profound respect for the surrounding environment
	I believe I should act with humility and respect
	I feel that is worth my earnest effort to experience and appreciate
	I realise that I am part of a greater existence or community.
Commandment	I have gained a clearer understanding of the life principles
	I feel that there are explicit rules should be strictly adhered to
	I have the opportunity to practice my moral and ethical beliefs
	I have certain responsibilities or obligations that I should fulfill

The selection of street view images perceived by volunteers was not based on the order of adjacent street view points but was extracted using an incomplete random method, allowing for a more comprehensive coverage of the study area [47]. Finally, all the Tibetan Buddhist perception rating data from volunteers were compiled to form the label dataset for the next step of random forest training. This method of creating perception labels, conducted entirely via the internet, is more convenient, efficient, and cost-effective compared to traditional survey methods.

### Urban street visual element data

To explain and predict mechanisms that may lead to a place’s perception associated with Tibetan Buddhism, we introduced visual element data to determine the connections between a place and the human perception of restorativeness. In order to interpret restorative perception through visual element data, we employed image semantic segmentation technology to calculate the proportion of semantic object elements in each streetscape image. Scene semantic parsing, one of the key techniques for understanding scene perception, aims to segment and identify object instances in a streetscape image. Given an input streetscape image, a trained model can predict visual element labels for each category. We trained a classic SegNet model [48], which demonstrated good scene segmentation performance, achieving an accuracy of 90.83% on the training set and 89.95% on the validation set. This level of accuracy enables effective interpretive work for restorative perception.

The training dataset for image semantic segmentation selected the Cityscapes dataset [49], which encompasses 34 categories of objects found in everyday life scenes, including the sky, roads, cars, and plants. Cityscapes is a dataset for semantic understanding of urban street scenes, primarily comprising street scenes from 50 different cities in Germany, such as Zurich, Hamburg, and Aachen. It includes 2,975 images for training, 500 for validation, and 1,525 for testing. The research utilized the dataset with 19 categories enabled by default for training purposes.

**Fig 4:**
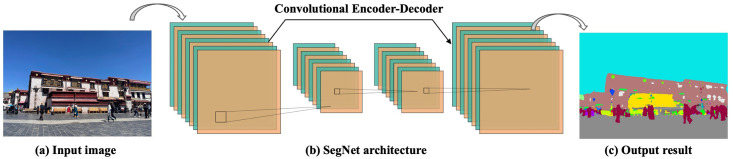
Overview of image semantic segmentation using SegNet trained on Cityscapes dataset. (a) Input urban natural image; (b) architecture of SegNet; (c) output image with a segmentation mask.

To train and implement the deep learning method in this study, and to standardize the computing variables for training, all training processes were conducted on the same Windows platform computer. This computer was equipped with an NVDIA GeForce GTX1070 graphics card, an AMD Ryzen5 2600X Six-Core Processor at 3.60GHz, and 16GB RAM. Fig 4 presents an overview of the semantic segmentation of street view images. The study utilized SegNet, an open-source project for image segmentation developed and released by a team from the University of Cambridge in 2015. This project allows for pixel-level precise segmentation of objects within images. The network comprises mainly two parts: an encoder and a decoder. The encoder primarily compresses and extracts information about the objects, while the decoder restores the extracted semantic information back to the input image size. This means each pixel can be classified and represented by the color corresponding to its object information.

In the neural network setup, the input image remapping window is set to 416×416 pixels, and the learning rate reduction method is set to decrease by 50% when there is no reduction in loss over 3 epochs. An early-stopping training method is employed, where training ceases and the network training completes if there is no decrease in loss over 10 epochs. During the transfer learning training phase, the Adam optimizer [50] is used with a learning rate set at 0.001. In the global learning training phase, the same Adam optimizer is utilized with a learning rate of 0.0001. Training data is recorded using the Tensorboard module included with Keras, taking 1 hour and 53 minutes for the transfer learning training phase and 4 hours and 41 minutes for the global learning training phase, totaling 6 hours and 34 minutes to complete the training. Our research team established the SegNet neural network using the Python language, Keras deep learning framework, transfer learning technology, and Tensorboard module, and recorded the training process. The code for replicating these experiments can be downloaded from our GitHub site at https://github.com/landscapewl/Segnet-Transfer-Learning.

## Methodology

As depicted in Fig 1, our research process comprises three parts. In this section, we primarily focus on the second part, which involves the use of a machine learning algorithm based on Random Forest to score the perception of Tibetan Buddhism in urban streets. This facilitates the acquisition of a comprehensive description of the urban streets’ Tibetan Buddhist perception. Additionally, the third part of our study utilizes the Point of Interest (POI) data model to explore the practical factors influencing these perceptions.

### Perception prediction of Tibetan Buddhism based on random forest algorithm

In order to explore urban residents’ perceptions of city streets, we employed a machine learning algorithm model based on the random forest method. This machine learning model has demonstrated good fitting aptitude in prior research practices [47, 51, 52], and we used it to conduct a convenient and effective assessment of the perception of urban streets in Lhasa. Each volunteer scored 50 street view images on the perception of Tibetan Buddhism, and the scores from all volunteers were compiled into a random forest training dataset. During the training process of the random forest model, two-thirds of the samples were randomly selected using bootstrap for data fitting or classification, while the remaining one-third, defined as out-of-bag (OOB) data, were used to evaluate the overall model error and the importance of variables. In the formula, when Xj is one of the input variables, to calculate the importance of the input variable Xj in the Nth tree VIn, it is necessary to use the sample data extracted by bootstrap to establish the regression tree model Tn, and then recalculate the OOB prediction error. Finally, randomly replace the observation values in variable Xj. Rebuild the model Tn’, and calculate the OOB’ prediction error. After processing the prediction errors of the two OOB datasets, the average result of all regression trees is the importance of variable Xj in the Nth random tree VIn(Xj), and the specific formula is as follows:

$$VI_{n}(X_{j})=\left\{\sum_{i=1}^{N_{\text{OOB}}}I\left[f(X_{i})=f_{n}(X_{i})\right]-\sum_{i=1}^{N_{\text{OOB}}}I\left[f(X_{i})=f_{n}\left(X_{i}^{\text{'}}\right)\right]\right\}/N_{OOB}$$ (1)

### Factors influencing the perception of Tibetan Buddhism

This study aims to explore the factors influencing the perception of Tibetan Buddhism in Lhasa from two aspects: the urban micro-environment and the city’s developmental status. The urban micro-environment is closely related to perception. We have collected data on the distribution of 150 types of urban micro-environment elements through semantic segmentation and identified the five most prevalent elements, namely Buildings View Index, Sky View Index, Green View Index, Roadway View Index, and Sidewalk View Index. Point of Interest (POI) data, provided by online map services, is a dataset that facilitates an intuitive and large-scale understanding of urban structure and function, and has been widely applied in urban studies, urban management, and social and cultural research. In this study, POI data was collected to represent the city’s developmental status. The POI dataset was retrieved using the API provided by Amap (https://lbs.amap.com/). POIs in the Amap database are categorized into over 20 types. We reclassified the original POI dataset into five categories to represent basic urban functions, namely Residential, Business, Commercial, Public Services, Entertainment, and Transport, making up six categories in total. After data cleaning and screening, we obtained 41,698 valid POI data entries (Table 2). Furthermore, we calculated the diversity and total number of POIs to better represent the city’s developmental status. The statistical characteristics of each factor influencing the perception of Tibetan Buddhism are shown in Table 3.

**Table 2:** Descriptive statistics of 6 types of Points of Interest (POIs).

POI types	Categories	Count	Percentage
Enterprise	Enterprise, company, factory	3874	9.29%
Commercial	Food, beverage, shopping mall, market, store, theatre, commercial service office	23886	57.28%
Residential	Commercial house, residential building,	870	2.09%
Entertainment	Scenic spot, park, open square, tourist attraction, gym, leisure place	1573	3.77%
Public service	Hospital, school, governmental organization, social group, management institution	10165	24.38%
Traffic	Bus stop, Subway station, Railway station	1330	3.19%

**Table 3:** Summary statistics for all variables of Tibetan Buddhist perception.

Variables (Unit)	Min.	Max.	Mean	SD
Urban visual built environment				
Building view index	0	0.529	0.325	0.111
Sky view index	0	0.074	0.001	0.004
Green view index	0	0.374	0.007	0.037
Person view index	0	0.298	0.009	0.023
Mountain view index	0	0.108	0.016	0.018
Urban development				
Enterprise POIs (N)	0	35	4.976	5.879
Commercial POIs (N)	0	462	144.767	70.831
Residential POIs (N)	0	10	1.818	2.258
Entertainment POIs (N)	0	32	2.304	4.263
Public service POIs (N)	0	201	18.274	27.099
Traffic POIs (N)	0	18	2.477	3.226

Note: Min. = Minimum; Max. = Maximum; SD = Standard deviation; N = Number.

### Tibetan Buddhism perception interpretation model

After calculating the perception scores of Tibetan Buddhism in Lhasa City, we examined the association between Tibetan Buddhism perception and influencing factors. Three different regression models were used, including Ordinary Least Squares (OLS) regression model, Spatial Lag Model (SLM), and Spatial Error Model (SEM).

OLS linear regression is a global model that estimates the parameters of explanatory variables in a linear model by minimizing the sum of squared differences between predicted and observed values in the dataset. The basic premise of the OLS model is that the residual terms are random and homoscedastic [53]. Its calculation formula can be expressed as Eq 1.


Y=Xβ+ϵ (2)

In this context, Y represents the dependent variable, X denotes the matrix of explanatory variables, β is the vector of coefficients, and ε signifies the vector of random error terms. However, when analysing spatial data, the Ordinary Least Squares (OLS) model may exhibit bias. This is primarily due to the frequent occurrence of spatial dependency in spatial data, meaning that the observed values at one location may depend on those of its neighbouring locations. To address this issue, this study further employed the Spatial Lag Model (SLM) and the Spatial Error Model (SEM) from structural equation modelling. The SLM posits that spatial dependency could result from autocorrelation of the dependent variable [54]. Its formula can be expressed as Eq 2:

$$Y=\rho \sum w_{ij}{\text{Y}}_{\text{j}}+\text{X}\beta +\epsilon$$ (3)

In this context, ρ represents the coefficient of spatial autocorrelation, Wij is the spatial weight between geographic units i and j, and other symbols are consistent with those in the OLS model.

The SEM tends to consider the autocorrelation of error terms [54]. Its calculation formula can be expressed as Eq 3.


$$Y=\text{X}\beta +\lambda \underset{\text{j}}{\sum }w_{ij}\epsilon_{\text{j}}+\epsilon$$ (4)

In this context, \(\lambda\) is the coefficient of spatial autocorrelation of errors, \(W_{ij}\) represents the spatial weight between geographical units \(i\) and \(j\), and other symbols are consistent with those used in the OLS model. In our statistical analysis, we use the cells of a fishing net (i.e., fishing net grid) as the basic spatial units and generate spatial weights using queen adjacency. Moran’s I analysis and spatial regression models are run in the GeoDa software [55]. It’s important to note that in this study, the fishing net serves both as a visualisation unit and as a unit of analysis.

## Results

### Spatial distribution characteristics of religious spiritual perception

**Fig 5:**
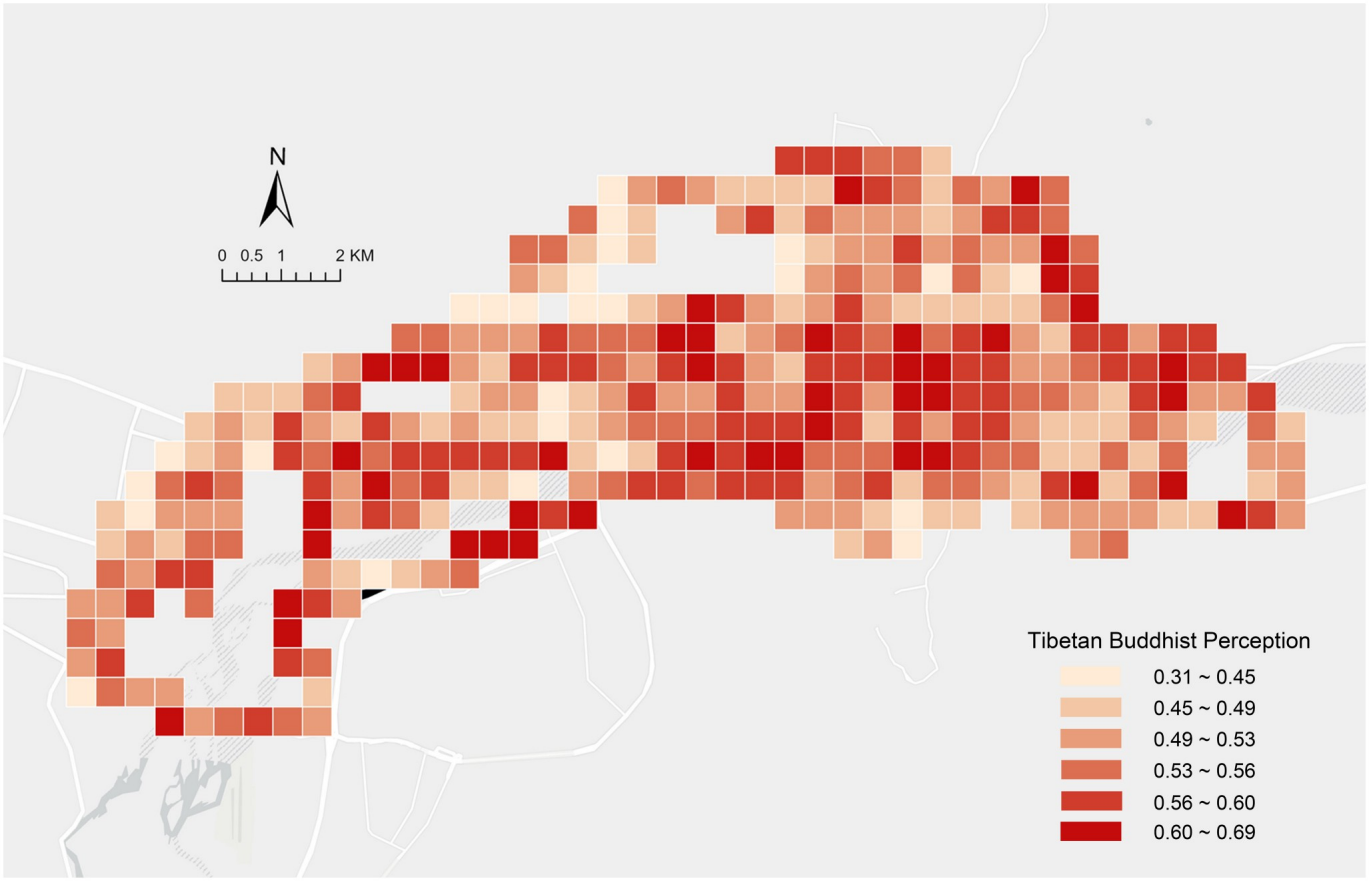
Spatial distribution characteristics of Tibetan Buddhist perception scores.

The research results were integrated into 500*500 meter fishing net units and visualized on a map (Fig 5). The perception scores of religious spirituality in the central urban area of Lhasa are relatively low, ranging from 0 to 0.69, with an average of 0.53. Overall, there is a noticeable spatial disparity in the distribution of scores for religious spiritual perception, with higher scores in central areas compared to peripheral regions, especially around temple buildings. Lhasa’s central urban area is more developed, while surrounding areas mostly comprise natural scenic spots and park green spaces. Religious spiritual symbols often attach themselves to buildings, landscape features, and signposts, thus showing a certain correlation with the degree of urban development. On the other hand, our results also demonstrate the spatial heterogeneity of religious spiritual perception in Lhasa. In areas with higher urban development, there are also many areas with low perception scores. This indicates that Lhasa, as the core area of Tibetan Buddhism, has an urbanization process similar to other cities, lacking attention to its own urban characteristics. This has led to a lack of protection and cultivation of the religious and cultural characteristics of Tibetan Buddhism in the construction process. Simultaneously, areas with high and medium scores are relatively concentrated, whereas areas with low scores are more dispersed.

### Distribution characteristics of religious spirit perception in space

**Fig 6:**
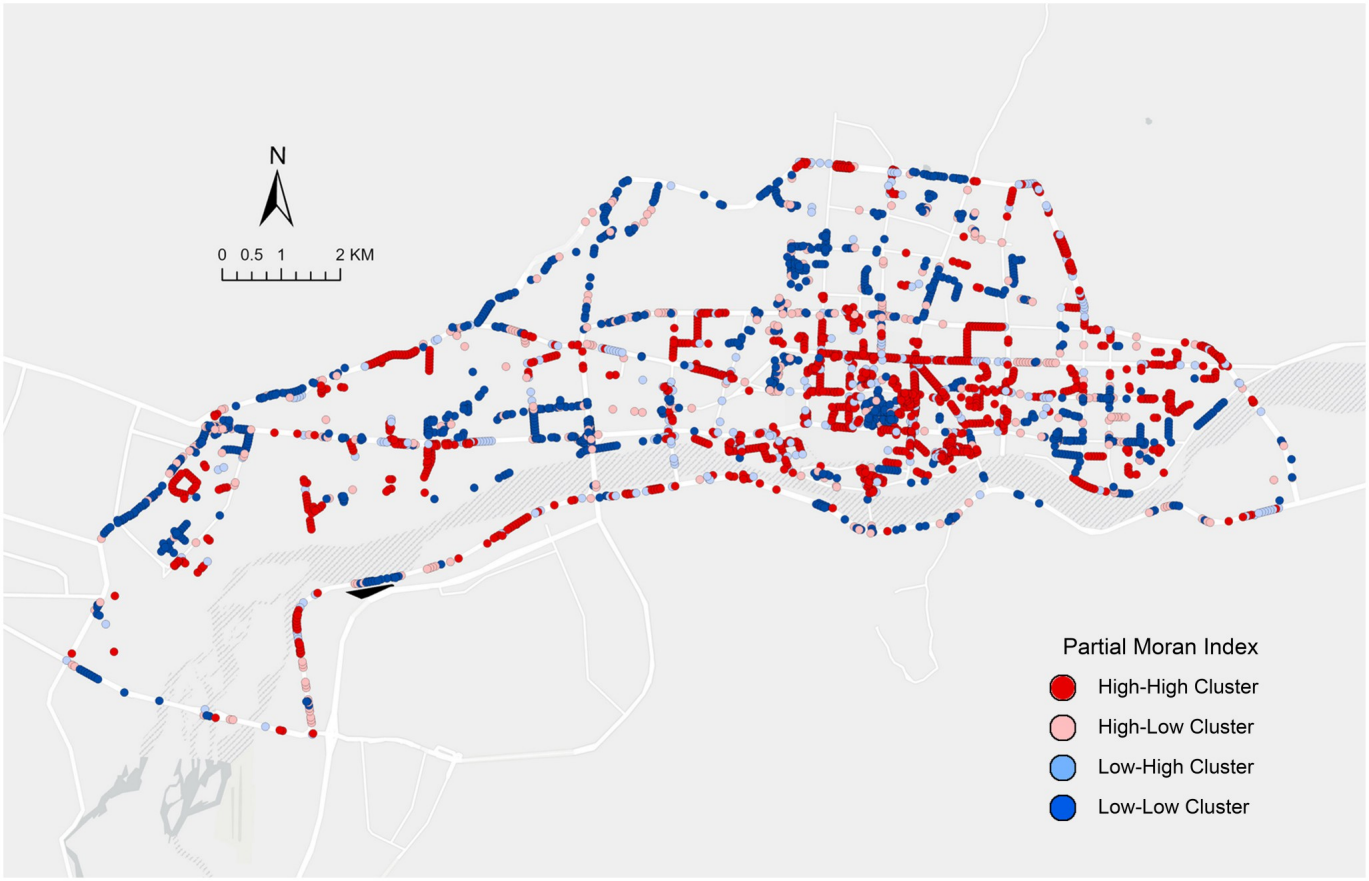
Tibetan Buddhism perception local autocorrelation spatial distribution map.

Fig 6 further visualizes the results of the local spatial autocorrelation analysis of religious spiritual perception by excluding streetscape points without spatial autocorrelation and categorizing points with spatial clustering relationships into four types: High-High Cluster, High-Low Cluster, Low-High Cluster, and Low-Low Cluster. The High-High Cluster is relatively densely distributed in the figure, particularly in the central and eastern areas. The central area, featuring the famous Tibetan Buddhist site, the Potala Palace, exhibits distinct characteristics of religious spiritual perception. The presence of High-High Cluster features in the eastern area indicates that this region has preserved its historical and cultural elements well, retaining significant Tibetan Buddhist characteristics. The Low-Low Cluster is more dispersed in the figure, with a higher concentration in peripheral urban areas. These peripheral areas, mostly natural scenic regions, lack the introduction of cultural elements, leading to an aggregation of low scores in religious spiritual perception. This dispersed distribution highlights the disruption of religious spiritual perception in the process of urban development, resulting in a lack of integrity in religious historical elements. The distribution of High-Low Cluster and Low-High Cluster is relatively scattered, indicating these locations have more complex interfering factors, which is common in developing cities. The distribution of High-Low Cluster shows some concentration around High-High Cluster areas, suggesting that the influencing elements of these two types of clusters have certain consistencies.

### Regression analysis result

Our study further revealed the association between Tibetan Buddhism perception and urban environmental elements through regression analysis. Prior to conducting the regression analysis, we used the Variance Inflation Factor (VIF) to test for multicollinearity among the independent variables. The results indicated that the VIFs of both the dependent and control variables were less than 7, suggesting the absence of multicollinearity issues and thus, ensuring the reliability of the regression results. The Ordinary Least Squares (OLS) model was conducted in Geoda, and the diagnostic results are shown in Table 3. The significant Koenker (BP) test indicates that the impact of various influencing factors is non-stationary in space; the same variables exhibit different fitting effects in different spatial regions, evidencing spatial heterogeneity. Therefore, it is necessary to further analyze using the Structural Equation Model (SEM). The diagnostic’s Lagrange Multiplier (LM) and Robust LM are commonly employed to identify the fit of spatial models (Table 4). The results showed that the Spatial Lag Model (SLM) had higher Lagrange Multiplier (SLM’s 79.130 vs. SEM’s 75.957) and Robust LM (SLM’s 3.358 vs. SEM’s 0.185) values than the SEM, indicating that the SLM is more suitable for explaining the association between Tibetan Buddhism perception and various influencing factors.

**Table 4:** OLS diagnostics of stress.

Indicator		Indicator	
**Number of observations**	369	**S.E. of regression**	0.057
**R-squared(R** ^**2**^ **)**	0.085	**Adjusted R-squared(R** ^**2**^ **)**	0.057
**Akaike info criterion(AIC)**	-1057.45	**Log likelihood value(LLV)**	540.726
**F-Statistic**	3.030	**Prob**	0.000***
**Breusch-Pagan test**	44.813	**Prob**	0.000***
**Koenker-Bassett test**	34.736	**Prob**	0.000***
**Lagrange Multiplier (lag)**	73.933	**Prob**	0.000***
**Robust LM (lag)**	10.702	**Prob**	0.001**
**Lagrange Multiplier (error)**	64.997	**Prob**	0.000***
**Robust LM (error)**	1.766	**Prob**	0.184

Note:

*** denotes significance at the 0.1% level,

** at the 1% level, and

* level 5% level.

To explore the spatial regression model that best explains the relationship between Tibetan Buddhism perception and urban environmental elements, we compared the performance of various models (Table 5). Based on the consideration of spatial heterogeneity in Tibetan Buddhism perception, both SLM and SEM showed a significant improvement over the OLS model in terms of model fit (R2). Lower AIC values and higher LLV values indicate superior model performance. In terms of fit, AIC, and LLV, SLM outperformed SEM and OLS. Therefore, our primary focus in this paper is on the results of the SLM.

**Table 5:** Results of regression models for GS exposure.

Variables	OLS		SLM		SEM	
	Coef.(SE)	P-Value	Coef.(SE)	P-Value	Coef.(SE)	P-Value
Urban visual built environment						
Buildings view index	-0.009 (0.035)	0.782	-0.019 (0.031)	0.455	-0.071 (0.039)	0.072*
Sky view index	2.041 (0.870)	<0.001***	1.728 (0.768)	<0.001***	1.505 (0.077)	<0.001***
Green view index	-0.001 (0.109)	0.996	-0.045 (0.097)	0.644	-0.112 (0.105)	0.288
Road view index	-36.832 (9.152)	<0.001***	-29.388 (3.457)	<0.001***	-20.892 (3.361)	<0.001***
Person view index	0.290 (0.013)	0.031*	0.293 (0.012)	0.012*	0.269 (0.059)	0.002**
Urban development						
Enterprise POIs	2.319×10^−4^ (6.398×10^−4^)	0.717	4.219×10^−4^ (5.650×10^−4^)	0.455	0.001 (0.001)	0.312
Commercial POIs	3.700×10^−5^ (1.189×10^−4^)	0.755	4.534×10^−5^ (1.050×10^−4^)	0.666	5.032×10^−5^ (1077×10^−4^)	0.640
Residential POIs	2.260×10^−4^ (0.002)	0.908	9.322×10^−5^ (0.002)	0.957	1.235×10^−4^ (0.001)	0.945
Entertainment POIs	0.001 (0.001)	0.006*	0.001 (0.001)	0.005**	0.001 (0.001)	0.005**
Public service POIs	-3.073×10^−6^ (3.248×10^−4^)	0.993	-2.095×10^−4^ (2.869×10^−4^)	0.465	-3.045×10^−4^ (3.022×10^−4^)	0.313
Traffic POIs	0.003 (0.001)	0.03**	0.002 (0.001)	0.006**	0.002 (0.001)	0.007**
R^2^	0.085		0.263		0.264	
LL	540.73		570.07		569.37	
AIC	-1057.45		-1114.15		-1114.75	

Note: Coef. = Coefficient; SE = Standard error; LL = Log-likelihood; AIC = Akaike information criterion.

*** denotes significance at the 0.1% level,

** at the 1% level, and

* level 5% level.

Regression results between religious spiritual perception and urban environmental factors show that Sky View Index significantly positively correlates in all models, while the Buildings View Index and Green View Index showed no significant correlation. The Road View Index exhibited a significant negative correlation across all models, and the Person View Index demonstrated some level of positive correlation in each model. Regarding urban development indicators, apart from Entertainment and Traffic Points of Interest (POIs) showing significant positive correlations in some models, other POIs did not exhibit significant correlations. Overall, the SLM and SEM models outperformed the OLS model in explanatory power and fit. These results reveal a complex relationship between urban visual built environments, urban development indicators, and Tibetan Buddhist perception, providing new insights into how urban environments influence cultural perceptions.

## Discussion

### Characteristics and importance of Tibetan Buddhist spiritual perceptual spaces

The core aim of this paper is to explore the specific manifestations and interactions of Tibetan Buddhist spiritual perceptual spaces in urban environments, using street view image recognition technology, combined with methodologies from human geography and cultural psychology [56]. Our in-depth research and analysis have revealed that these spiritual perceptual spaces possess multidimensionality, fluidity and adaptability, as well as symbolism and codification. These spaces are influenced by various factors including physical space, social structure, and cultural customs, and exhibit strong adaptability and fluidity in the face of urban transformation and cultural evolution. Moreover, these spiritual perceptual spaces have profound impacts on individual and community psychological health, cultural heritage, social cohesion, and sustainable development [57]. Particularly, as vital carriers of cultural and historical legacy, they play an indispensable role in shaping individual and community identities. Therefore, spiritual perceptual spaces hold significant importance in modern urban development and management. Their protection and rational utilisation not only contribute to cultural inheritance and social harmony but are also integral to achieving sustainable urban development [58]. This paper aims to highlight these research findings, advocating for greater academic, social, and policy attention towards spiritual perceptual spaces, and promoting their effective protection and optimisation in the modern urban context.

From the regression analysis results of this paper, it can be seen that the urban visual built environment, such as sky perspective and road perspective index, has a significant impact on Tibetan Buddhist spiritual perception of space. This highlights the importance of considering the visual environment of the city when protecting and optimizing the mental perception space. For example, a significant negative correlation in the road perspective index suggests that excessive urbanization and road construction may adversely affect these Spaces. Therefore, urban planning should give more consideration to how to integrate and protect these spiritual Spaces in order to promote a more harmonious urban ecology.

### The philosophical theory foundation and extensibility of the method

When discussing the spatial characteristics of Tibetan Buddhism, it is essential to consider its emphasis on a cosmological view, harmony, and the philosophical essence of spiritual enlightenment, as well as its architectural forms [59]. Tibetan Buddhist architecture often employs symbolically rich elements and patterns representing Buddhist doctrines and cosmology. The spatial layout of these buildings is designed to facilitate community gatherings and individual meditation, reflecting Buddhist concepts of community and personal growth. Moreover, these structures often integrate seamlessly with the natural landscape, mirroring the Tibetan Buddhist pursuit of harmonious coexistence with nature. This interplay between philosophy and architecture highlights the profound connection between spiritual beliefs and their material expression [60]. This is also the core basis for our ability to identify and map the spatial characteristics of Tibetan Buddhism through visual landscapes.

Although Tibetan Buddhism originated from Indian Buddhism [61], it has developed more regional uniqueness and distinctiveness, as evidenced in its strict disciplinary rules and extensive integration with the daily lives of residents [62]. As the heartland of Tibetan Buddhism, the urban layout and architectural style of Lhasa are deeply influenced by Tibetan Buddhism. Temples and monasteries in Lhasa typically adopt traditional Tibetan architectural styles, blending Buddhist elements with local cultural characteristics [63]. The urban space of Lhasa is not only the center of religious activities but also the core of Tibetan cultural and social life. The ideas of Tibetan Buddhism are reflected in every aspect of the lives of residents in Tibetan areas, showing consistency in residential patterns, architectural forms, materials used, and courtyard construction [64]. Especially, Tibetan Buddhist religious style buildings have maintained unique architectural forms and spiritual symbols through a long history and still retain complete functionality for use by the Tibetan people today, continuing to influence Tibetan people. The spatial distribution characteristics of Tibetan Buddhist spiritual places in Lhasa and the general trends observed in the urban environment may indicate similar patterns in other Tibetan areas, allowing the methods used in this study to be applied and promoted in other towns in Tibetan areas.

Additionally, our proposed method of studying religious spiritual spaces based on subjective perception can also be applied in other typical religiously influenced areas around the world, such as Hàndì, Kathmandu, and Kyoto. The Buddhism in Hàndì is primarily Theravada, characterized by a more functional and simplistic architectural style. In Kathmandu, the coexistence of Buddhism and Hinduism has led to a mixed architectural style. Here, Buddhist temples often incorporate local Nepalese architectural features, such as multi-tiered roofs and intricate wood carvings. The urban layout of Kyoto reflects a unique Japanese aesthetic of symmetry and sequence, closely linked to the layout of its Buddhist temples, reflecting a more holistic and unified planning concept. The degree of urbanization and modernization in these areas also affects how traditional spiritual practices and spaces are preserved and integrated into the urban structure, necessitating careful observation in these regions.

### Precise identification and protection of spiritual spaces

Based on the research of this article, we propose the following management and policy recommendations for more effectively identifying and protecting the spiritual perception spaces of Tibetan Buddhism. Firstly, it is essential to explicitly incorporate the protection of spiritual perception spaces in urban planning and development policies, and to establish related protection indicators and assessment mechanisms. Secondly, the use of street view image recognition technology and community participatory surveys should be considered to comprehensively identify and assess the current status and socio-cultural value of these spiritual places. This approach would not only generate more accurate spatial data but also enhance community residents’ awareness and appreciation of these sites. Thirdly, we suggest promoting public education and cultural outreach activities to increase public understanding of the importance of spiritual perception spaces, thereby forming a societal consensus and motivation for protection. For example, a variety of methods such as exhibitions, lectures, and community events could be employed to educate more people about the cultural and spiritual significance of these places. Finally, we recommend that governments and relevant institutions establish specific funding and support mechanisms to encourage more research and practical activities, fostering effective interaction and cooperation between scientific research and social practice. This would not only aid in our deeper understanding and protection of these places of significant cultural and spiritual value but also promote sustainable urban development and social harmony.

### Recommendations for future research

Based on the research context and main findings of this paper, there are several directions and methodologies that future research could explore. Firstly, further in-depth studies can be conducted on the roles and functions of different types of spiritual perceptual spaces—such as temples, meditation venues, and cultural heritage centres—in various urban environments. Secondly, although this paper primarily utilised street view image recognition technology, future research could consider employing a more diverse range of data collection methods, such as drone aerial photography, three-dimensional modelling, or virtual reality technology, to obtain more comprehensive and detailed spatial information. Thirdly, in addition to studying Tibetan Buddhism’s spiritual perceptual spaces, research could be extended to other religious or cultural traditions for broader and more diverse comparative studies. Fourthly, future research could attempt to employ advanced technologies like big data and machine learning for more precise and systematic quantitative analysis of these spiritual perceptual spaces. This would not only provide a more scientific basis for policy-making but also aid in methodological innovation in the study of spiritual spaces. Lastly, it is recommended to conduct more interdisciplinary collaborative research, integrating theories and methods from multiple fields such as psychology, sociology, and environmental science into the study of spiritual perceptual spaces, to foster a more comprehensive and profound understanding. These are all directions and methodologies worthy of further exploration and development in future research.

## Conclusion

Our research examined the spatial identification of Tibetan Buddhist spiritual places in Lhasa, finding that these spaces are notably concentrated in areas rich in natural and cultural heritage. This underscores the intrinsic connection between Tibetan Buddhism and its natural environment, highlighting the resilience of spiritual practices amidst urbanization. Utilizing street view data and deep learning, we demonstrated the potential of digital tools in mapping and understanding the impact of urban development on spiritual and cultural landscapes. The results of this study reveal the manifestation of Tibetan Buddhism in urban spaces, emphasizing the importance of considering specific cultural and religious traditions in urban planning and the preservation of ancient buildings. These findings are crucial for urban planning, religious studies, and digital humanities. Our research advocates for incorporating spaces of spiritual perception into urban development strategies, emphasizing their role in cultural heritage preservation and sustainable urban development. The adaptability of Tibetan Buddhist spiritual places in the process of modernization highlights their significance as cultural and spiritual pillars, continuing to influence the material spatial layout in Tibetan areas. In summary, this study paves the way for future interdisciplinary research on spaces of spiritual perception across cultures and cities. It stresses the necessity of balancing material, cultural, and spiritual elements in urban development, ensuring the sustainability and cultural richness of urban growth. Moreover, the analytical methods used in this study, employing street view data and deep learning technology, can be applied to other cities globally with unique cultural and religious backgrounds, helping studyers and urban planners better understand and plan their own cultural and religious spaces.
